# Systemic Regulators of Skeletal Muscle Regeneration in Obesity

**DOI:** 10.3389/fendo.2017.00029

**Published:** 2017-02-16

**Authors:** Indranil Sinha, Dharaniya Sakthivel, David E. Varon

**Affiliations:** ^1^Division of Plastic Surgery, Brigham and Women’s Hospital, Boston, MA, USA

**Keywords:** skeletal muscle, obesity, inflammation, metabolism, insulin

## Abstract

Skeletal muscle maintenance is a dynamic process and undergoes constant repair and regeneration. However, skeletal muscle regenerative capacity declines in obesity. In this review, we focus on obesity-associated changes in inflammation, metabolism, and impaired insulin signaling, which are pathologically dysregulated and ultimately result in a loss of muscle mass and function. In addition, we examine the relationships between skeletal muscle, liver, and visceral adipose tissue in an obese state.

## Introduction

Current estimates are that one-third of the United States population is obese, and this number is rapidly escalating (1). Many of these patients additionally suffer from preclinical or overt type 2 diabetes mellitus (T2DM) (1, 2). Multiple studies suggest that skeletal muscle wasting in these patients, especially those above the age of 60 years, can be severe (3–5). Diminished capacity for skeletal muscle regeneration likely contributes to the loss of lean muscle mass seen in diabetic patients (6). Obesity, a common precursor to T2DM, is also noted to have significant and independent negative effects on lean skeletal muscle mass (7). This is correlated with insulin resistance and reduced muscle performance (8). Overall, these patients suffer from a significant decline in muscle strength, as compared to age-matched controls, and a loss of functional independence (3–5). However, the effects of obesity on skeletal muscle regeneration remain largely unknown. Stimulation or preservation of skeletal muscle regeneration could possibly enable these patients to improve their strength and functional activity, as well as maintain skeletal muscle mass (8, 9).

Recent studies demonstrate that mice fed a high-fat diet (HFD) exhibit reduced hind limb muscle mass and form fewer and smaller fibers following skeletal muscle injury (10). Additionally, there exists a reduction in the total number of satellite cells, which are required for skeletal muscle regeneration (10, 11). Therefore, it is of significant clinical importance to understand how obesity impacts muscle regeneration and identify mechanisms that may be targeted for therapeutic benefit. Skeletal muscle mass in these patients is not only essential for ambulation but also necessary for glucose utilization and maintaining insulin sensitivity (12). Multiple factors affect muscle mass in patients with obesity including satellite cell function, inflammation, insulin signaling, and metabolic derangements. Furthermore, obesity-related increases in visceral adipose tissue (VAT) and fatty acid accumulation in the liver, as with non-alcoholic fatty liver disease (NAFLD), are intimately linked to the maintenance of muscle mass. When evaluating obesity and corollary loss of skeletal muscle mass, systemic mediators and their effect on muscle regeneration must be considered.

## Satellite Cells

Satellite cells in skeletal muscle are located beneath the basal lamina of mature muscle fibers and are thought to be the major source of regeneration following muscle injury (13). It is now known that the satellite cell population is heterogeneous and contains both myogenic and non-myogenic cell populations. Using fluorescence-activated cell sorting (FACS), unique, myogenic stem cells, or skeletal muscle precursors (SMPs), within the satellite cell pool can be identified and isolated for further study (14). Early life obesity, induced by HFD, results in both a reduction in SMP cell frequency and impaired differentiation (10). Specifically, myogenic differentiation (MyoD), a critical factor in promoting skeletal muscle differentiation, is significantly reduced in satellite cells isolated in a diet-induced obesity (DIO) murine model (15).

In addition, satellite cell activation in a murine DIO model is impaired, which can be partially attributed to a loss in hepatocyte growth factor (HGF) signaling in skeletal muscle (16). Skeletal muscle-specific decrease in active HGF following injury limits activation of satellite cells from their quiescent state. HGF activation of SMPs requires AMP-activated protein kinase (AMPK), a protein essential to maintain satellite cell number and induce myotube formation. The active, or phosphorylated form of AMPK, promotes skeletal muscle glucose uptake and increases insulin sensitivity. Recent studies suggest that the satellite cells isolated from injured muscles of DIO mice demonstrate decreased AMPK activity and impaired regeneration (17). Fibrogenic/adipogenic precursors (FAPs) are a separate and distinct population in the satellite cell compartment (18). These cells are unable to directly form myofibers but can promote the differentiation of SMPs or form adipose tissue based on the local environment (18–20). A common observation in conditions associated with impaired skeletal muscle insulin sensitivity is an accumulation of ectopic lipids within (*intracellular*) and between (*extracellular*) skeletal muscle fibers (21), which is linked to reduced insulin sensitivity and diminished muscle function (22). The contribution of FAPs to these intramuscular lipid deposits remains unknown, as does their precise contribution to skeletal muscle regeneration in a model of obesity.

## Inflammation

Obesity results in chronic, low-grade skeletal muscle inflammation (23). Recent studies further suggest DIO alone can reprogram both skeletal muscle and liver to increase the production of proinflammatory cytokines, including tumor necrosis factor-alpha (TNF-α), interleukin 1-beta (IL-1β), and IL-6 (24). Increased IL-6 has been shown to limit skeletal muscle differentiation *in vitro* (25). In murine models of cachexia, both IL-6 or nuclear factor-kappa B (NF-κB) overexpression in skeletal muscle causes severe muscular atrophy (26, 27). Separately, in persistent inflammatory conditions, IL-6 actions are associated with increased muscle wasting (28). Despite multiple studies suggesting that skeletal muscle-specific upregulation of proinflammatory cytokines induces muscle wasting; this area warrants further research in regard to obesity. To date, no studies have shown that IL-6 or NF-κB inhibition, either systemically or in skeletal muscle, improves regeneration in an obesity model. In addition, it remains unclear whether local inflammation from skeletal muscle, increased cytokine release from liver or visceral fat, or a combination are required for impaired muscle regeneration and loss of muscle mass.

TNF-α, another proinflammatory cytokine, also has catabolic effects on muscles in chronic inflammatory state. Elevated TNF-α circulation in obese models can cause muscle wasting, inflammatory myopathies, and insulin resistance by regulating activation and secretion of other proinflammatory cytokines (29, 30). TNF-α supplementation additionally limits C2C12 muscle myoblast cell differentiation *in vitro* by repressing MyoD synthesis. The effects of TNF-α on skeletal muscle regeneration in an obese model remains unknown.

In addition, obesity further promotes deposition of macrophages in VAT, which contributes to inflammation, increased lipolysis, and subsequently ectopic fat deposition in skeletal muscles (31). In the early stages of obesity, an increase in these macrophages precedes T cell accumulation. T cells, in turn, are polarized into proinflammatory Th1 cells that cause myocyte inflammation through interferon secretion. The inhibition of ectopic macrophage accumulation in fat may reverse insulin resistance and thereby improve muscle function (32, 33). These points further highlight that muscle wasting in obesity is a systemic issue, instead of secondary to local changes in skeletal muscle alone.

## Insulin Resistance

An array of growth factor signaling cascades, regulated by insulin, are required for the proper maintenance of skeletal muscle mass. Obesity-associated insulin resistance alters these pathways and can variably inhibit muscle regeneration. Insulin signaling is a highly complex pathway within skeletal muscle, mediated by insulin growth factor-1 (IGF-1) (34). Specifically, downstream of IGF-1, both the mitogen-activated protein kinase (MAPK) and phosphatidylinositol-4,5-bisphosphate 3-kinase (PI3K) pathways are known to regulate skeletal muscle regeneration (35).

Mitogen-activated protein kinases are enzymes that become catalytically activated in response to diverse stimuli such as mitogens, osmotic stress, and proinflammatory cytokines. MAPK activity mediates the crosstalk between canonical and non-canonical transforming growth factor (TGF-β) in a DIO model (36). In skeletal muscle, TGF-β1 inhibits differentiation of fetal myoblasts (37). Separately, increased levels of TGF-β can cause muscle injury to heal with fibrosis, rather than regenerated skeletal muscle (38). Increased p38 MAPK and TGF-β activity within ectopic adipocytes may induce satellite cell senescence (39). Paradoxically, results from C2C12 studies, a murine myoblast model for skeletal muscle development, demonstrates a positive role for activated MAPK in cell migration (40). MAPK signaling and activity remain controversial with respect to skeletal muscle regeneration in obesity, and this topic warrants further research. Interestingly, follistatin supplementation improves muscle growth in circumstances with elevated TGF-β signaling (41).

In models of muscular dystrophy, an increase in PI3K activity can be beneficial for regeneration, as it increases Akt activity and downstream, promyogenic factors, which stimulate muscle growth. Akt activation also helps in preventing muscle atrophy by inducing the expression of mammalian target of rapamycin and ribosomal protein S6 kinase beta-1 (S6K1) (42). Specifically, in DIO models, an increase in Akt activity by phosphatase and tensin homolog (PTEN) inhibition restores skeletal muscle regeneration (11). The role of decreased insulin signaling with regard to skeletal muscle injury remains a topic of active research.

## Metabolism

Obesity and chronic overnutrition are closely associated with increased mitochondrial-derived oxidative stress (43, 44). Skeletal muscle from obese or diabetic patients shows decreased mitochondrial content and a corollary loss of fatty acid oxidation (45, 46) associated with excess caloric consumption and non-inherent mitochondrial dysfunction (47). In patients with T2DM, targeted overexpression of catalase within mitochondria can protect skeletal muscle from ischemic injury, but the role of oxidative stress and mitochondrial dysfunction in obesity-related loss of skeletal muscle regeneration remains unknown (48, 49). In the context of obesity, skeletal muscle undergoes a protective shift to retain its functional capacity by converting to glycolytic, type II muscle fibers, mediated by Brg1/Brm-associated factor (Baf60c) (50–52). The Baf60c pathway increases Akt activation, which, as discussed previously, improves diet-based glucose tolerance and increases insulin sensitivity. Independently, muscle-specific Akt activation also leads to hypertrophy of type II muscle fibers with subsequent resolution of hepatic steatosis, decreased fat mass, and improved metabolic parameters (53). However, Baf60c signaling is decreased in obese rodent models, possibly due to the inhibitory effects of TNF-α (51, 52).

In contrast, many studies suggest that hypertrophy of oxidative muscle fibers (type I) can also promote metabolic homeostasis. Muscle-specific overexpression of peroxisome proliferator-activated receptor-delta (PPAR-δ) (54, 55) promotes higher levels of type I fibers relative to type II fibers, improved performance in endurance, exercise, and resistance to DIO. Conversely, mice that are deficient in peroxisome proliferator-activated receptor-gamma (PPAR-γ) coactivator 1-alpha (PGC-1α) display abnormal oxidative fiber growth and develop an increase in body fat (56). These studies suggest that increased energy expenditure in skeletal muscle mediated by hypertrophy can protect against weight gain and metabolic dysfunction. In addition, myostatin-deficient mice are resistant to DIO (57), but this metabolic effect may be due to either changes in type I or type II fibers, or from the direct action of myostatin on adipose tissue (58). Overall, it remains unclear whether or not a type I or type II fiber majority contributes to the improvement in metabolic parameters in DIO, but an increase in muscle mass, in general, appears to counteract the metabolic derangements seen in obesity.

In the absence of muscle hypertrophy, reactive oxygen species (ROS) accumulate in skeletal muscle. In obese conditions, increased ROS production is associated with contractile dysfunction, chronic oxidative stress followed by protein loss, and muscle atrophy (59). ROS is also capable of modulating the insulin signaling pathway, although the exact mechanism remains unclear. Studies suggest that ROS decreases insulin response and contributes to impaired mitochondrial activity (60). Sirtuin (SIRT), a NAD(+)-dependent histone deacetylase (HDAC) localized in mitochondria, has been found to regulate several mitochondrial genes and is important in muscle differentiation, activation of myogenesis, and skeletal muscle metabolism. Specifically, SIRT1 promotes glycolysis and inhibits adipogenesis, thereby attenuating obesity-related insulin resistance (61–63). Conversely, in T2DM, inhibition of SIRT1 alters mitochondrial metabolism and increases the production of ROS (64).

Histone deacetylases, in general, are a group of enzymes that regulate gene expression by altering chromatin structure. In obesity models, HDAC inhibition restores PPAR-γ function improving skeletal muscle glucose and fatty acid metabolism. HDAC inhibition also generates non-traditional effects such as reducing adipose tissue expansion, resistance to obesity, and improvement in insulin sensitivity (65, 66). HDAC inhibitors have proven their potency in hampering fibrosis and favorably encouraging therapeutic muscle regeneration (67). Evaluation of HDAC inhibitors for the treatment of obesity-related muscle wasting is underway (68).

In skeletal muscle, glucose transporter 4 (GLUT4) levels are directly associated with increased oxidative capacity (69). Increases in GLUT4 translocation to the plasma membrane promotes improved rates of satellite cell proliferation and differentiation (70). AMPK increases GLUT4 gene expression in human skeletal muscles (71). AMPK is also a widely recognized regulator of energy metabolism. Decreased AMPK activity is associated with metabolic disorders such as obesity and T2DM (18, 72). AMPK also plays a key role in upregulating the transcription levels of paired box protein 7 (Pax7), myogenic factor 5, myogenin, and MyoD, all of which are necessary for muscle growth. Although metabolic rate is stimulated through AMPK activity, ATP/AMP ratios for the AMPK activation pathways are not affected by obesity (18, 73).

Skeletal muscle isolated from patients with T2DM shows reduced levels of diacylglycerol kinase-delta (DGKδ), a key enzyme in triglyceride biosynthesis required for appropriate AMPK function. DGKs control the expression levels of diacylglycerol (DAG) by catalyzing its conversion to phosphatidic acid utilizing ATP (74). Elevated plasma free fatty acid (FFA) levels from enlarged adipose tissue in obese models force intramyocellular DAG accumulation (75). In an obese population, increased DAG accumulation, secondary to reduced DGK or increased, circulating FFA, results in inhibition of both glucose uptake and glycogen synthesis. This further exacerbates insulin resistance.

## Liver and Fat

**Figure 1:**
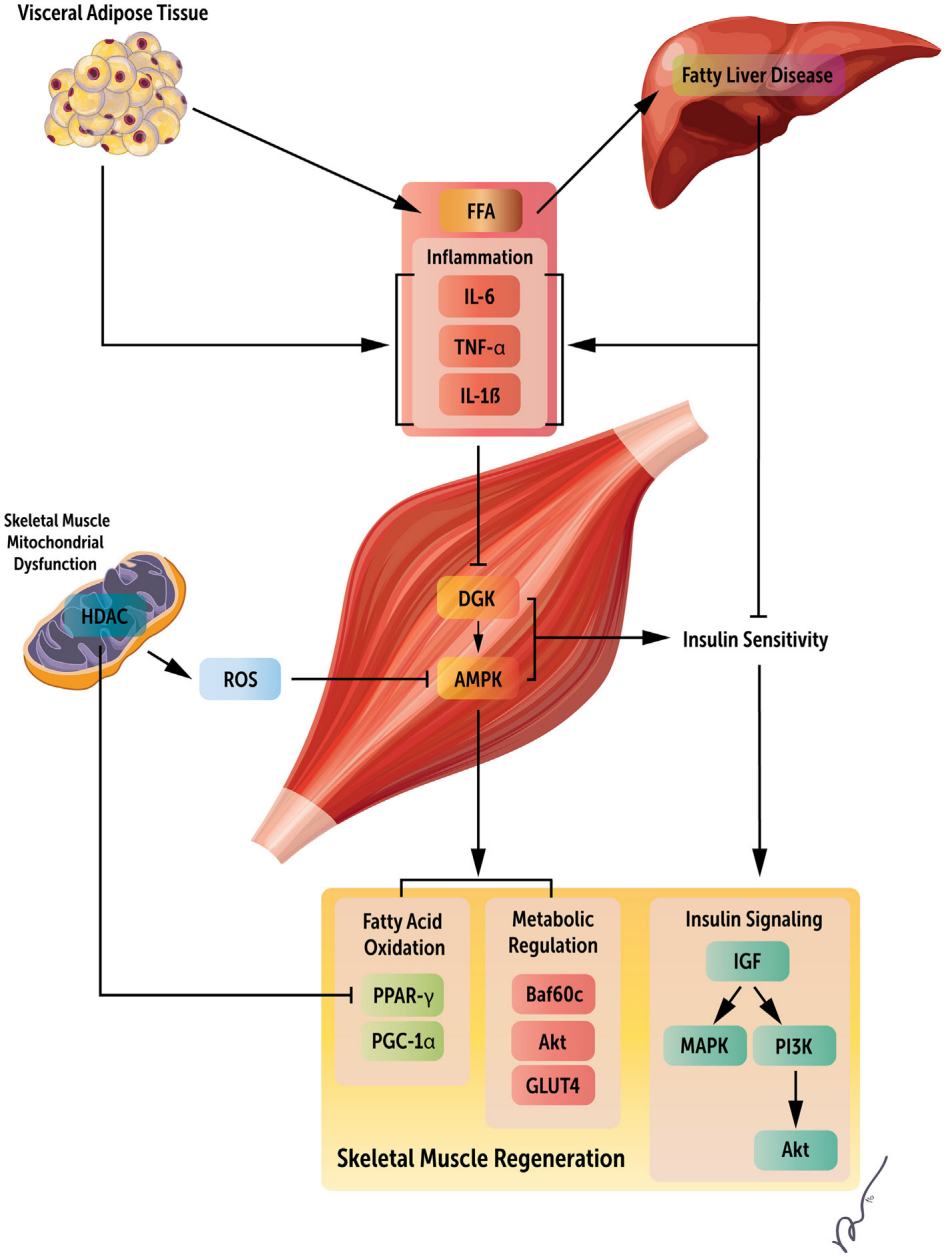
**Systemic regulators of obesity mediated loss of skeletal muscle regeneration**. Obesity results in both increased visceral adipose tissue and fatty acid accumulation in the liver. These changes manifest as increased circulating fatty acids, inflammatory mediators, and insulin resistance, leading to metabolic derangements within skeletal muscle, and ultimately, decreased skeletal muscle regeneration by the deregulation of multiple signaling pathways. This figure summarizes key factors limiting muscle regeneration in an obese state.

As previously noted, obesity-associated liver dysfunction can have a profound impact on skeletal muscle maintenance and regeneration. NAFLD commonly occurs in obesity and is correlated with sarcopenia, even in the absence of insulin resistance (76, 77). Loss of muscle mass reduces a key cellular target for insulin action, contributing to glucose intolerance and, in turn, further muscle depletion. In addition, NAFLD is associated with the production of multiple proinflammatory factors, including NF-κB, IL-6, and TNF-α, all of which are known to be protein catabolic (78). VAT also releases circulating FFA, leading to further liver damage (79). Independently, VAT can also result in higher levels of proinflammatory cytokines, similar to the liver (80). Thereby, liver damage and VAT accumulation work synergistically to impair skeletal muscle regeneration in obesity by increasing FFA circulation, proinflammatory cytokines, and limiting promyogenic insulin actions on muscle. These pathways are depicted in Figure 1.

## Conclusion and Perspectives

Obesity is accompanied by significant health concerns, including severe loss of skeletal muscle mass. The maintenance of skeletal muscle is necessary for ambulation, proper insulin signaling, and glucose homeostasis. Obesity-related loss of muscle mass perpetuates a cycle of increasing metabolic abnormality, associated liver dysfunction, and further muscle loss. Effective methods to target obesity-associated muscle wasting must account for multiple systemic changes that occur, including increased inflammatory mediators, circulating FFA, metabolic dysfunction, and insulin resistance. Further research is warranted to determine specific molecular mechanisms that limit skeletal muscle regeneration and induce atrophy in an obese state.

## Author Contributions

IS and DS drafted the manuscript and DV developed the figure. All the authors contributed to its intellectual content and critical revisions.

## Conflict of Interest Statement

The authors declare that the research was conducted in the absence of any commercial or financial relationships that could be construed as a potential conflict of interest.
